# Skeletal phenotypes in postmenopausal women affected by primary hyperparathyroidism

**DOI:** 10.3389/fendo.2024.1475147

**Published:** 2024-10-29

**Authors:** Sabrina Corbetta, Laura Gianotti, Elena Castellano, Silvia Carrara, Francesca Raineri, Elisabetta Munari, Gregorio Guabello, Elisa Cairoli, Iacopo Chiodini, Luca Giovanelli, Laura Pierotti, Elisa Dinoi, Simone Della Valentina, Filomena Cetani

**Affiliations:** ^1^ Bone Metabolism Disorders and Diabetes Unit, Istituto di Ricerca e Cura a Carattere Scientifico (IRCCS) Istituto Auxologico Italiano, Milan, Italy; ^2^ Department of Biomedical, Surgical and Dental Sciences, University of Milan, Milan, Italy; ^3^ Division of Endocrinology and Diabetology, Department of Medicine, Azienda Sanitaria Locale Cuneo 1 (ASL CN1), Cuneo, Italy; ^4^ Division of Endocrinology, Diabetology and Metabolism, Department of Internal Medicine, Santi Croce & Carle Hospital, Cuneo, Italy; ^5^ Department of Medical Biotechnology and Translational Medicine, University of Milan, Milan, Italy; ^6^ Service of Bone Metabolism, Istituto di Ricerca e Cura a Carattere Scientifico (IRCCS) Ospedale Galeazzi Sant’Ambrogio, Milan, Italy; ^7^ Unit of Endocrinology, Aziende Socio Sanitaria Territoriale (ASST) Ospedale Niguarda, Milan, Italy; ^8^ Department of Clinical and Experimental Medicine, University of Pisa, Pisa, Italy; ^9^ Endocrine Unit 2, University Hospital of Pisa, Pisa, Italy

**Keywords:** osteoporosis, fractures, primary hyperparathyroidism, PTH, hypercalcemia

## Abstract

**Purpose:**

The current primary hyperparathyroidism (PHPT) presents as a mild disease. We explored skeletal phenotypes in postmenopausal women affected by PHPT, focusing on fracture prevalence.

**Methods:**

PHPT women were retrospectively evaluated at four Italian centers for osteoporosis management (two centers in Milan, *n* = 244; Cuneo, *n* = 128; Pisa, *n* = 131). Data collected from clinical records were analyzed by hierarchical clusterization.

**Results:**

Considering the whole PHPT series [*n* = 503, aged 67.0 (61.0–74.0) years], 90% had low bone mineral density (BMD) and approximately 30% reported at least one fracture. Vertebral fractures were associated with older age and lower hypophosphatemia, while women with appendicular fractures were younger with less severe hypophosphatemia. Fractures were predicted by lumbar *T*-score. By using a clustering approach, we identified four different skeletal phenotypes (cluster, C): C1 (*n* = 53) and C2 (*n* = 172) included women with lumbar and femur neck osteopenia, with low prevalence of fractures (11.3%). Osteoporotic PHPT women were grouped into C3 (*n* = 142) and C4 (*n* = 136); all women in C4 experienced fractures, were older, and were more frequently affected with cardiovascular diseases. In contrast, women included in C3 never experienced fractures and had a lower body mass index (BMI), though they were characterized by severe reduction in BMD at both lumbar and femur sites. Ionized and total calcium, phosphate, 25hydroxyvitamin D levels, kidney function, and stone prevalence (range, 26.4%–29.0%) were similar among clusters C1, C2, and C4, while unfractured women in C3 showed slightly higher ionized hypercalcemia, lower hypophosphatemia, and higher hypercalciuria with a trend to more frequently develop kidney stones (38.7%) than women in the remaining clusters.

**Conclusions:**

Skeletal involvement in women with PHPT presented heterogeneous phenotypes with different prevalence of fractures. Fractures were not related to PHPT severity, suggesting that other factors besides PHPT, such as age, BMI, and lumbar and femur BMD, should be considered in the evaluation of bone involvement in postmenopausal women with PHPT.

## Introduction

The current most common clinical presentation of primary hyperparathyroidism (PHPT) is characterized by mild disease, where classical bone involvement of osteitis fibrosa cystica is absent, but osteopenia/osteoporosis is frequent (1). PTH promotes bone resorption and bone formation, and therefore, bone is a main target of PHPT. Assessment of bone mineral density (BMD) by dual x-ray absorptiometry (DXA) in PHPT patients demonstrated preferential reduction of BMD at sites with predominantly cortical bone such as the radius, with relative sparing of sites with predominantly trabecular bone such as the lumbar spine. Moreover, PHPT has been associated with impaired trabecular microarchitecture evaluated by trabecular bone score (TBS) and reduced bone material strength index (2).

The skeletal manifestations of PHPT are low BMD and a high bone turnover state with increased propensity to fracture. A recent meta-analysis showed that the risk of vertebral fractures (VFs) was tripled in PHPT patients, the risk of forearm fractures was doubled, while the risk of femur fracture (FF) was similar compared to controls (3). The risk of VF was even further exacerbated in subgroups of studies that included mild PHPT patients (i.e., those with no complications from PHPT), and that included postmenopausal women (3). Similarly, PHPT patients showed an increased prevalence of both major and hip fractures at the time of PHPT diagnosis compared with the general population, and an increased risk of major and hip fractures at follow-up, irrespectively to conservative versus surgical treatment (4). These recent studies investigated skeletal aspects in PHPT disease occurring throughout the lifespan.

Indeed, PHPT predominantly occurs in postmenopausal women, who have an increased risk of osteoporosis and fractures due to aging and loss of the protective effects of estrogen (5); it is conceivable that PHPT further increases this risk in postmenopausal women. The present study explored the skeletal phenotype at the time of PHPT diagnosis in a series including exclusively postmenopausal women affected with a wide clinical and biochemical spectrum of PHPT. The study aimed (1) to investigate the skeletal phenotype of PHPT in postmenopausal estrogen-deficient condition and (2) to identify clinical and/or metabolic factors associated with the occurrence of fractures in postmenopausal PHPT women.

## Patients and methods

### Patients

The diagnosis of PHPT was based on increased ionized or albumin-adjusted serum calcium and increased or inappropriately normal intact PTH levels (6).

A total of 503 patients were enrolled in the study. All patients were managed at four tertiary care referral Italian Endocrine Centers for the management of bone and mineral diseases during the period 2013–2023: IRCCS Ospedale Galeazzi Sant’Ambrogio and IRCCS Istituto Auxologico Italiano in Milan (*n* = 244), AO Universitaria Pisana in Pisa (*n* = 131), and AO Santi Croce & Carle in Cuneo (*n* = 128).

Inclusion criteria were as follows: (1) women with symptomatic and asymptomatic PHPT according to recent guidelines (6), and (2) menopause at the time of PHPT diagnosis and from at least 5 years from last menses.

Exclusion criteria were as follows: (1) male gender; (2) less than 5 years from the last menses; (3) clinical and/or genetic diagnosis of familial hypocalciuric hypercalcemia (HHC, OMIM 145980, 145981, and 600740); (4) familial PHPT (MEN1, OMIM 131100; MEN2, OMIM 171400; MEN4, OMIM 610755; HPT-JT, and OMIM 145001); (5) parathyroid carcinoma; (6) ionized calcium levels <1.28 mmol/L (i.e., diagnosis of normocalcemic PHPT); (7) diseases (with the exception of diabetes) or therapies affecting the bone metabolism; (8) ongoing (previous 6 months) antiresorptive treatment; (9) lack of DXA measurement at lumbar or hip sites; and (10) DXA not performed by a Hologic or Lunar densitometer.

### Study design

This is a retrospective study considering data collected at the time of PHPT diagnosis; the following data were collected in all enrolled patients: (1) BMD assessment by DXA (Hologic or Lunar) at lumbar and total hip and femoral neck; (2) detection of clinical and morphometric fractures by conventional thoracic and lumbar x-rays (see specific paragraph for details); (3) collection of previous hip, pelvic, ankle, humerus, and wrist fragility fractures; (4) concomitant comorbidities (diabetes, arterial blood hypertension, and others) and ongoing medical treatment; (5) anthropometric data (weight and height) and calculation of body mass index (BMI); (6) occurrence of symptomatic or asymptomatic kidney stones; (7) circulating ionized and total calcium, phosphate, creatinine, 25hydroxyvitamin D (25OHD), and parathormone (PTH); (8) 24-h urinary calcium measurement; and (9) circulating bone markers (total and bone alkaline phosphatase, ALP and BSAP, respectively).

All procedures performed in studies involving human participants were in accordance with the ethical standards of the institutional and/or national research committee and with the 1964 Helsinki Declaration and its later amendments or comparable ethical standards. Ethical approval was waived by the local Ethics Committee of each participating center in view of the retrospective nature of the study. and all the procedures being performed were part of the routine care. All patients gave their informed consent and data were anonymously collected in a database.

### Biochemistry

All blood samples were obtained after overnight fasting and rest. The collection of 24-h urine was obtained under free diet conditions and treated as calcium excretion per kilogram of body weight.

Serum total calcium, phosphate, creatinine, ALP, and 24-h urinary calcium were analyzed by a standard autoanalyzer using colorimetric and enzymatic methods, whereas ionized serum calcium was analyzed by an ion-selective electrode method after correction for pH. Plasma PTH was measured by a second- or third-generation assay among the different centers; thus, it was treated as fold change of the upper limit of the normal range for each specific assay. Serum 25OHD was measured by chemiluminescent immunoassay. BSAP was determined by monoclonal immunoenzymatic assay.

Urine calcium excretion was routinely measured in 24-h urine collection under free diet conditions and treated as calcium excretion per kilogram of body weight. The estimated glomerular filtration rate (eGFR) was calculated using the EPI-CKD (Chronic Kidney Disease Epidemiology Collaboration) formula (7) in each patient.

### Bone mineral density measurements

BMD was measured at the lumbar spine (L1–L4), proximal femur, using the following instruments: DXA QDR-4500 (Hologic, Bedford, MA) or Lunar iDXA (GE Healthcare). BMD was expressed as *T*-scores (difference from the mean BMD value of healthy young people in SD units).

According to World Health Organization (WHO) recommendations, osteoporosis and osteopenia were defined as a *T*-score value ≤ −2.5 SD and <−1 and >−2.5, respectively.

### Fracture assessment

Vertebral fractures: the presence of VF s was assessed in all patients at diagnosis by conventional thoracic and lumbar x-rays at the Milan and Cuneo Units, while at the Pisa Unit, VFs were assessed by DXA morphometry and, when positive, confirmed by x-rays. Vertebrae were classified as normal (<25% reduction) or fractured (>25% reduction in vertebral body height, i.e., moderate deformity according the Genant semiquantitative grading scale for VF s) (8). X-rays were graded by visual inspection of two trained reviewers at each center.

Nonvertebral fractures: major low-trauma fractures (femur, proximal humerus, or wrist) were recorded in all subjects by medical history. In case of fractures, an expert radiologist reviewed the x-ray images.

### Statistical analysis

Data were collected from clinical records and analyzed by Spearman correlation test corrected for multiple correlations to identify parameters significantly correlated with fractures. Multiple regression analysis using a model including age at diagnosis, lumbar *T*-score, femur neck *T*-score, ionized calcium, PTH fold change, phosphate, and creatinine indicated that fractures were predicted by lumbar and femur neck *T*-scores; all other variables were not predictive.

*T*-scores at lumbar and femur neck sites and number of fractures detected in each enrolled PHPT woman were considered as criteria for performing unsupervised hierarchical clusterization by using Wards’ method and Euclidean similarity index. Hierarchical clusterization creates groups so that patients within a group are similar to each other and allows to identify differences from patients in other groups. This technique can group patients with similar symptoms or medical histories, aiding in diagnosis and care quality.

Data were compared among the identified clusters by Kruskal–Wallis ANOVA analysis corrected for multiple comparisons (Dunn’s test) or ordinary one-way ANOVA corrected for multiple comparisons (Holm-Sidak’s test) as appropriate.

Nonparametric data failing normality test was expressed as median and interquartile range; normally distributed data were expressed as mean ± standard deviation. Statistical analysis was performed by Past 4. A value of *p* less than 0.05 was considered as statistically significant.

The size of the series allowed us to detect differences between clusters with a *post-hoc* power (1 − β error) of 0.99 and an α error of 0.05 and an effect size of 0.58 by G power.

## Results

### Clinical features of postmenopausal PHPT women

The enrolled 503 postmenopausal PHPT women aged 67.0 (61.0–74.0) years with a median age at menopause of 49.7 (48.0–52.0) years. Their median BMI at diagnosis was 25.9 (22.5–28.3) kg/m^2^. The PHPT women included from the four tertiary care centers in Italy had similar age at diagnosis, BMI, and prevalence of hypertension and diabetes. The three series differed in some clinical and biochemical features reflecting the specific clinical setting (**Supplementary Table 1**): the Milan series included PHPT women with a milder biochemical and hormonal disease and a higher prevalence of kidney involvement; the Cuneo series included women with lower 25OHD levels, while PHPT women in the Pisa series less frequently experienced fragility fractures. According to the current guidelines (6), 58% of the enrolled PHPT women were asymptomatic.

### Prevalence of low bone mineral density and fragility fractures

Osteoporosis, with a *T*-score of less than −2.5 with or without fractures, was diagnosed in 239 women (47.5%) at the lumbar site and in 172 women (34.2%) at the femur site, while osteopenia, with a *T*-score ranging from −1.0 to −2.5 with or without fractures, was diagnosed in 190 women (37.8%) at the lumbar site and in 277 women (55.1%) at the femur site. Osteoporosis at any site was diagnosed in 320 women (63.6%).

At least one fragility fracture at any skeletal segment was detected in 149 out of 503 (29.6%) PHPT women for a total of 302 fractures; 37 out of 183 (20%) osteopenic women and 112 out of 320 (35%) osteoporotic women experienced fractures.

VF s occurred in 80 women (53.7% of fractured patients), experiencing from one to six VFs; FFs were reported by 18 women (12.0% of fractured patients), while appendicular (wrist, humerus, tibial, rotula, and ankle) fractures (AFs) were detected in 66 women (44.3% of fractured patients). Of note, women reporting wrist fractures represented the 29.5% of fractured women. Multiple fractures were experienced by nearly half of fractured PHPT women (*n* = 70, 47%); similarly, more than a half of PHPT women (60.0%) with FF experienced multiple fragility fractures.

PHPT women with prevalent VF (*n* = 80) were older at the time of diagnosis of PHPT compared with unfractured patients, similar to the median age of women with FF (**Table 1**). Median BMD *T*-scores were significantly lower at the lumbar and femur neck sites in PHPT women with VF or FF compared with those without fractures. Non-vertebral non- FFs, mainly AF, not associated with VF and/or FF, were detected in 66 PHPT women. PHPT women with AF did not differ from unfractured women in terms of BMD *T*-score at any site, while they had significantly higher serum phosphate levels (**Table 1**).

**Table 1:** Comparison between fractured and unfractured PHPT postmenopausal women according to different types of fragility fractures.

Parameter	Vertebral FX	Femur FX	Appendicular FX	NO FX	*p*
*N*	80	18	66	353	
Age (years)	72.0 (63.0, 78.0)*	73.0 (66.0, 79.0)*	66.0 (61.0, 72.2)	65.0 (60.0, 73.0)	**0.0005**
BMI (kg/m^2^)	25.0 (23.0, 28.9)	24.0 (22.3, 28.5)	24.0 (21.5, 27.1)	25.1 (22.6, 28.3)	0.4879
Mineral metabolism					
25OHD (ng/mL)	32.2 (26.1, 40.9)	32.0 (16.7, 34.0)	31.7 (23.1, 38.1)	30.2 (22.0, 38.6)	0.4559
PTH fold change	1.82 (1.33, 2.82)	1.52 (1.32, 2.09)	1.68 (1.36, 2.54)	1.81 (1.39, 2.54)	0.4892
Ionized Ca^2+^ (mmol/L)	1.42 (1.37, 1.51)	1.39 (1.36, 1.56)	1.39 (1.34, 1.45)	1.42 (1.36, 1.48)	0.1533
Total Ca^2+^ (mg/dL)	10.9 (10.4, 11.4)	11.1 (10.4, 11.4)	10.8 (10.4, 11.2)	10.8 (10.4, 11.3)	0.9011
Phosphate (mg/dL)	2.7 (2.4, 3.1)	2.9 (2.5, 3.2)	2.9 (2.6, 3.2)*	2.7 (2.4, 3.0)	**0.0132**
Kidney involvement					
Urine Ca^2+^ (mg/24 h)	283.5 (192.5, 386.3)	211.0 (156.0, 340.0)	262.0 (196.5, 361.5)	300.5 (197.5, 394.1)	0.3945
Urine Ca^2+^ (mg/kg/24 h)	4.7 (2.8, 6.6)	4.1 (2.0, 4.9)	4.5 (2.8, 4.9)	4.6 (3.1, 6.2)	0.3514
eGFR (mL/min)	85.0 (66.5, 93.6)	77.1 (63.5, 88.7)	85.1 (74.8, 92.0)	85.6 (72.4, 95.0)	0.4623
Kidney stones (%)	30.0	22.0	28.8	32.3	0.7757
Bone involvement					
ALP (U/L)	90.0 (73.0, 111.5)	108.0 (66.0, 115.5)	96.5 (71.2, 115.5)	88.0 (68.0, 113.0)	0.9453
BASP (ng/mL)	21.5 (13.7, 31.0)	18.0 (11.9, 30.0)	24.2 (12.8, 33.8)	24.0 (15.0, 35.1)	0.3959
Lumbar *T*-score	−2.90 (−3.70, −1.88)*	−3.30 (−4.15, −2.10)*	−2.60 (−3.30, −2.00)	−2.40 (−3.20, −1.50)	**0.0012**
Femoral neck *T*-score	−2.50 (−3.10, −1.90)*	−2.50 (−3.23, −2.05)	−2.40 (−2.93, −1.88)	−2.10 (−2.70, −1.50)	**0.0032**

Each skeletal segment involved in at least one fracture was considered as a single event for the analysis.

Data are presented as median and interquartile range and analyzed by Kruskal–Wallis ANOVA corrected for multiple comparisons (Dunn’s test).

*p < 0.05 vs. NO FX. n, number of fragility fractures; BMD, bone mineral density; BMI, body mass index; 25OHD, 25hydroxyvitamin D; PTH, parathormone; Ca^2+^, calcium; eGFR, estimated glomerular filtration rate; ALP, alkaline phosphatase; BSAP, bone-specific alkaline phosphatase. Bold values indicate statistically significant values.

### Mineral metabolism markers and vitamin D status

Most women (*n* = 405, 80.5%) displayed mild PHPT, defined as serum total calcium <1.0 mg/dL, the upper limit of the normal range (9). Moreover, serum total calcium lower than 10.0 mg/dL was detected in 28 (5.6%) PHPT women, though all had plasma ionized calcium levels higher than 1.28 mmol/L. Circulating PTH levels ranged from 0.608- to 19. 138- fold, the upper limit of the normal range. Circulating 25OHD levels higher than 30 ng/mL at the time of diagnosis were detected in 262 (52.1%) PHPT women, while 18.0% had 25OHD levels lower than 20 ng/mL, and in particular, 8.2% had 25OHD levels lower than 12 ng/mL (10). Therefore, most PHPT women had sufficient vitamin D at diagnosis.

### Parameters correlating with fractures and bone mineral density

The number of prevalent fractures positively correlated with age at diagnosis, and negatively correlated with femoral neck, total hip, and lumbar *T*-scores (**Table 2**). Lumbar and femoral neck *T*-scores positively correlated with BMI. Of note, lumbar and neck *T*-scores negatively correlated with PTH levels, as well as with ionized and total calcium. Moreover, lumbar *T*-scores negatively correlated with kidney calcium excretion, eGFR, and total ALP levels, while both femoral neck and lumbar *T*-scores positively correlated with serum phosphate levels (**Table 2**).

**Table 2:** Correlations by Spearman with fragility fractures, lumbar, hip BMD *T*-scores, and clinical and biochemical parameters in the cohort of PHPT postmenopausal women.

Parameters	*R*	*p*
Fractures (*n*)		
Age at diagnosis	0.159	<0.0001*
Lumbar *T*-scores	−0.146	**0.0010**
Femoral neck *T*-scores	−0.165	0.0002*
Hip *T*-scores	−0.216	<0.0001*
Lumbar BMD *T*-scores		
BMI	0.319	<0.0001*
PTH (fold change)	−0.124	**0.005**
Ionized calcium	−0.081	0.071
Total calcium	−0.117	**0.009**
Urine calcium	−0.152	0.001*
Phosphate	0.101	**0.026**
eGFR	−0.145	**0.002**
Total ALP	−0.139	**0.006**
Femur neck BMD *T*-scores		
Age at diagnosis	−0.171	<0.0001*
BMI	0.350	<0.0001*
25OHD	−0.094	**0.0355**
PTH (fold change)	−0.089	**0.0448**
Ionized calcium	−0.139	**0.0018**
Total calcium	−0.100	**0.0253**
Phosphate	0.104	**0.0222**

Statistically significant or approaching the significance correlations have been reported.

*n*, number of fragility fractures; BMD, bone mineral density; BMI, body mass index; PTH, parathormone; eGFR, estimated glomerular filtration rate; ALP, alkaline phosphatase; 25OHD, 25hydroxyvitamin D. *Statistically significant correlations after Bonferroni adjustment. Bold values indicate statistically significant values.

Multiple regression analysis indicated that fractures were predicted by lumbar and neck *T*-scores, but not by age at PHPT diagnosis (Wilks’ lambda 0.966, *p* = 0.0005 by MANOVA).

### Cluster analysis

Based on the results of multiple regression analysis, clusterization considering lumbar *T*-scores, femur neck *T*-scores, and fractures identified four clusters (**Supplementary Figure 1**). Fractures explained 54% of variance, while the second component of variance was determined by neck *T*-score (31%). The subgroups were named cluster 1 (*n* = 53), cluster 2 (*n* = 172), cluster 3 (*n* = 142), and cluster 4 (*n* = 136), identifying four different bone phenotypes (**Table 3**).

**Table 3:** Comparison among the different phenotyping clusters.

Parameter	Cluster 1	Cluster 2	Cluster 3	Cluster 4	*p*
*N*	53	172	142	136	
Age (years)	65.0 (62.0, 73.5)	66.0 (59.0, 73.0)	65.0 (61.0, 73.0)	70.0 (63.0, 78.0)**,***	**0.0101**
BMI (kg/m^2^)	28.8 (25.0, 32.4)	26.0 (23.3, 28.4)*,***	23.7 (21.2, 26.8)*,**	24.4 (21.6, 28.2)*,**,***	**<0.0001**
Mineral metabolism					
25OHD (ng/mL)	28.0 (22.0, 35.5)	29.0 (22.0, 36.7)	33.0 (22.0, 41.0)	32.0 (23.8, 38.9)	0.1260
PTH fold change	1.70 (1.24, 2.33)	1.77 (1.40, 2.32)	2.04 (1.46, 3.24)	1.68 (1.35, 2.66)	**0.0360**
Ionized Ca^2+^ (mmol/L)	1.44 (1.38, 1.49)	1.40 (1.35, 1.46)	1.42 (1.37, 1.52)**	1.41 (1.36, 1.50)	**0.0229**
Total Ca^2+^ (mg/dL)	10.8 (10.6, 11.3)	10.8 (10.4, 11.1)	10.9 (10.5, 11.5)	10.9 (10.4, 11.3)	0.0830
Phosphate (mg/dL)	2.7 (2.2, 3.1)	2.8 (2.5, 3.1)	2.6 (2.2, 3.0)**	2.8 (2.5, 3.1)	**0.0142**
Kidney involvement					
Urine Ca^2+^ (mg/24 h)	236.5 (148.9, 331.0)	300.0 (196.0, 379.5)	332.0 (224.5, 436.5)*	274.0 (199.3, 372.3)	**0.0065**
Urine Ca^2+^ (mg/kg/24 h)	3.00 (2.02, 4.83)	4.40 (3.07, 5.61)	5.54 (3.88, 7.63)*,**	4.50 (2.89, 6.10)*,**,***	**<0.0001**
eGFR (mL/min)	77.0 (65.9, 91.4)	84.8 (74.3, 95.0)	87.6 (74.5, 97.0)*	85.0 (72.4, 92.2)	**0.0225**
Kidney stones (%)	26.4	29.0	38.7	28.7	0.1667
Bone involvement					
Total ALP (U/L)	86.0 (67.0, 108.0)	85.0 (65.0, 105.0)	92.0 (77.0, 116.0)	92.0 (72.0, 114.5)	0.0708
Lumbar *T*-score	0.0 (−0.40, 0.50)	−2.00 (−2.40, −1.50)*	−3.40 (−3.90, −3.00)*,**	−2.80 (−3.70, −2.20)*,**,***	**<0.0001**
Neck *T*-score	−1.30 (−2.00, −0.80)	−1.80 (−2.30, −1.30)	−2.80 (−3.40, −2.10)*,**	−2.50 (−3.08, −2.00)*,**,***	**<0.0001**
Fractures (*n*, min–max)	0.0 (0.0, 2.0)	0.0 (0.0, 1.0)	0.0 (0.0, 1.0)	1.0 (1.0, 7.0)*,***	**<0.0001**
Fractured (%)	11.3	3.5	0.7	100	**<0.0001**
VF (%)	5.7	1.2	0.0	55.9	**<0.0001**
FF (%)	1.9	0.0	0.0	10.3	**<0.0001**
AF (%)	5.7	2.3	0.7	35.3	**<0.0001**
Comorbidities					
Hypertension (%)	58.5	50.6	40.1	47.8	0.0979
Dyslipidemia (%)	56.8	46.5	44.6	39.0	0.2554
Diabetes (%)	15.1	7.6	6.3	6.6	0.1997
CVD (%)	4.5	12.3	10.0	17.0	**<0.0001**
Neoplasia (%)	18.2	16.2	12.0	19.0	0.5514
Others (%)	22.7	36.2	25.0	35.0	0.1336

Data were analysed by Kruskal–Wallis ANOVA for nonparametric data corrected by Dunn’s test for multiple comparison.

*p < 0.05 vs. cluster 1; **p < 0.05 vs. cluster 2; ***p < 0.05 vs. cluster 3. *n*, number of fragility fractures; BMD, bone mineral density; BMI, body mass index; 25OHD, 25hydroxyvitamin D; PTH, parathormone; eGFR, estimated glomerular filtration rate; ALP, alkaline phosphatase; min–max, minimum and maximum; VF, vertebral fracture; FF, femur fracture; AF, appendicular fracture; CVD, cardiovascular disease. Bold values indicate statistically significant values.

PHPT women included in cluster 1 had median femur neck osteopenia and low prevalence of fractures (11.3%) and differ from women included in the other clusters as they were heavier. Women in cluster 2 had lumbar and femur neck osteopenia and a very low prevalence of fractures. Median lumbar and femur neck diagnostics for osteoporosis characterized PHPT women grouped in clusters 3 and 4: cluster 3 included women with very low BMDs, all having never experienced fractures, while cluster 4 included women all having reported at least one fracture. Cluster 4 fractured women were older than women included in the remaining clusters. Of note, unfractured osteoporotic women had higher plasma ionized calcium, lower phosphatemia, and higher renal calcium excretion than unfractured osteopenic women (**Table 3**).

Women grouped in the four clusters showed similar prevalence of hypertension, dyslipidemia, diabetes, neoplasia, and other comorbidities, while osteoporotic fractured women in cluster 4 were more often affected with cardiovascular diseases (coronaropathy, heart failure, and cerebrovascular disease) than women in the remaining clusters (**Table 3**).

## Discussion

The study investigated the skeletal involvement in a large Italian series of postmenopausal women with PHPT focusing on the occurrence of fragility fractures. More than 80% of women suffered from mild PHPT with hypercalcemia within 1 mg/dL above the upper limit of the normal range (9), the most common clinical form of presentation of modern PHPT. Though mild clinical features, approximately 90% of postmenopausal PHPT women presented low BMD at the lumbar and/or femur neck site and nearly 30% reported at least one fragility fracture at the time of PHPT diagnosis. Therefore, according to the recent guidelines (6), two-thirds of PHPT postmenopausal women in the present series suffered from osteoporosis and should be considered as affected with asymptomatic disease with target organ involvement and surgical indication.

Data of the literature about prevalence and incidence of fractures in PHPT patients considered wide cohorts including both male and female patients of any age. A recent large Scottish population-based study with a mean follow-up of 8.8 years demonstrated that PHPT was associated with an increased risk of osteoporosis (HR = 1.31; 95% CI, 1.16–1.49) (11). Similarly, a Swedish cohort study revealed that PHPT is associated with increased risk of any fractures (HR 1.39; 95% CI 1.31–1.48) as well as hip fracture (HR 1.51; 95% CI 1.35–1.70) (12). Danish patients with PHPT had been found with a higher risk of hip fracture (+48%), major osteoporotic fracture (+36%), and also death (+52%) than population controls; at any given age, average 10-year probabilities of fracture were higher in patients with PHPT than population controls (4). During a mean follow-up of more than 5 years, the unadjusted incidence of fracture was 13.7% in United States PHPT patients observed without surgery (13). However, although fracture risk is increased in PHPT patients, the effect of successful parathyroidectomy on fracture incidence is controversial. Some data suggest a lower risk of any fracture and hip fracture among surgically treated older adults with PHPT (13), while randomized controlled trials reported no evidence of adverse effects of observation for at least a decade with respect to fractures in mild PHPT patients (4, 14–18).

In the present study, we focused the attention on postmenopausal women with PHPT, in which coexisting estrogen deficiency independently increases bone fragility.

Considering the sites of prevalent fractures in postmenopausal PHPT women, it emerges that VFs were associated with older age and lower serum phosphate levels, while AFs were associated with younger age, as observed in unfractured PHPT women, and associated with less severe hypophosphatemia. Hypophosphatemia has been recently reported to be associated with a higher risk of osteoporosis and renal stones in PHPT patients (19). This clinical landscape may support PHPT involvement in VF risk, while other undefined factors, excluding age and estrogen deficiency, determined AFs.

The clustering approach identified four main skeletal phenotypes. Of note, the circulating mineral metabolic profile was similar among the skeletal phenotypes, a finding in line with the conclusion of the Denmark register-based survey stating that the severity of the PHPT disease as judged by baseline serum calcium or PTH had no effect on fracture risk (4).

Moreover, osteopenic women, who were heavier than the osteoporotic ones, experienced fractures with low prevalence. Osteoporotic women were characterized by higher urine calcium excretion, with any significant difference between fractured and unfractured patients, similar to what was reported in normoparathyroid postmenopausal osteoporotic women (20). Considering women included in the osteoporotic clusters, a high risk of fractures emerges, which occurred in approximately 50% of women. Osteoporotic women experiencing fractures were older than women included in the other clusters and were more frequently diagnosed with cardiovascular diseases. This may be just an association of two frequent aging-related diseases, though interrelationships between bone and cardiovascular diseases are emerging (21).

The remaining unfractured osteoporotic women were younger, aging similar to the osteopenic women, though they presented very low lumbar and femur neck bone mineral densities; moreover, they were less heavy. Though their PHPT was mild, they showed slightly higher plasma ionized calcium levels, lower phosphatemia, and higher PTH levels, suggesting a more severe activity of the PHPT disease (19, 22, 23); nonetheless, they did not experience fractures. This is an unexpected paradox difficult to explain in the setting of the present study. Phosphate has been partially explored as a potential independent factor in determining bone fractures. Results from large prospective observational trials in two population-based cohorts, the Dutch Rotterdam Study and the US Osteoporotic Fractures in Men study, showed that phosphate levels were positively associated with fracture risk in men and women; in both studies, phosphate levels were within the normal range (24). Though it has been reported that hypophosphatemia positively correlated with BMD at cortical bone sites in PHPT patients, no data about association with fractures are available. In our series of PHPT-related hypophosphatemic women, severity of phosphatemia was not associated with an increased risk of fracture.

We are tempted to speculate that (1) older (>65 years old) PHPT osteoporotic women may be considered at high risk of fracture and antiosteoporotic therapy should be provided; (2) fractured osteopenic PHPT patients were likely to benefit of extensive diagnostic workup investigating for any further causes of bone fragility; and (3) younger and overweight PHPT women and without weight-adjusted hypercalciuria may be considered at low risk of fractures and, therefore, will likely experience poor improvement of their risk of fracture after successful parathyroidectomy.

The strengths of our study are as follows: (1) a large cohort of postmenopausal women with PHPT; (2) recruitment from different clinical settings, which ensured the inclusion of all hypercalcemic PHPT clinical spectrum; (3) all enrolled patients were followed at endocrine centers expert in bone metabolism in which patients were consistently and closely evaluated; and (4) clinical fractures were systematically recorded and vertebral morphometry was performed in all patients at the time of PHPT diagnosis.

Admittedly, the present study suffered from limitations: (1) the retrospective design of the study prevented the identification of predictive factors; (2) we were not able to define the duration of PHPT before the diagnosis; (3) data about BMD at distal radius as well as about TBS or other parameters of bone quality were not available; it has been reported that including the distal radius in BMD measurement increases the number of patients diagnosed with osteoporosis and for whom surgery may be indicated; circulating calcium and PTH levels were also more frequently elevated in patients with forearm osteoporosis (25); (4) PHPT women with previous anti-osteoporotic treatment were included; (5) PHPT women were evaluated in the presence of different vitamin D status, although we investigated vitamin D-sufficient PHPT women as more than 80% of PHPT women who had serum 25OHD levels above 20 ng/mL at the time of diagnosis and more than half had serum 25OHD levels higher than 30 ng/mL. Furthermore, Soto-Pedre et al. reported that the increased risk of fractures in PHPT patients was independent from the vitamin D status (11).

## Conclusions

Low bone density occurs in most postmenopausal PHPT women and one-third suffered from at least one fracture at the time of PHPT diagnosis; fractures occur in both osteopenic and osteoporotic PHPT women, although they are more frequent in older osteoporotic women. VF may be promoted by PHPT activity as suggested by concomitant more severe hypophosphatemia. There is a subset of postmenopausal PHPT women who never experienced fractures associated with low bone turnover and PTH levels, suggestive of poor active hyperparathyroid states. In these PHPT women, parathyroid surgery should not be proposed to reduce fracture risk. However, bone density and fractures are independent from PHPT severity defined by circulating calcium and PTH levels. These findings suggest that the evaluation of bone involvement in the specific setting of postmenopausal PHPT women to define the best therapeutic strategy (observation, medical therapy, and surgery) could not rely only on BMD evaluation and PHPT severity as defined by serum calcium and PTH levels, but it should take into consideration other factors such as age and weight of the patients, comorbidities, and concurrent therapies.

## Data Availability

The datasets presented in this study can be found in online repositories. The names of the repository/repositories and accession number(s) can be found below: 10.5281/zenodo.11077572.
