# Supplementary figures and images for: Skeletal phenotypes in postmenopausal women affected by primary hyperparathyroidism
Source: Front Endocrinol (Lausanne). 2024 Oct 29;15:1475147. doi: 10.3389/fendo.2024.1475147 (PMC11558525; doi:10.3389/fendo.2024.1475147)

## Slide 1
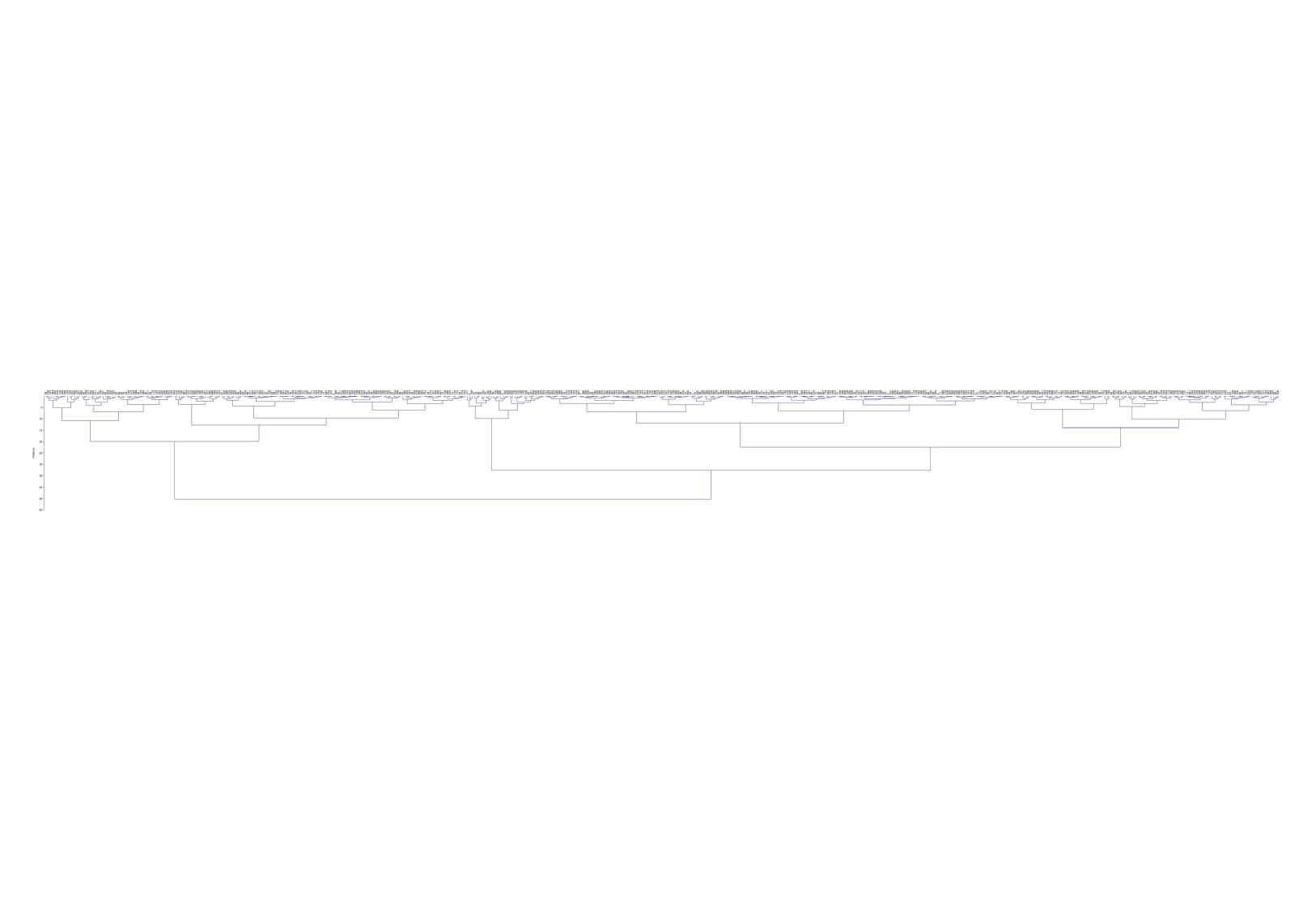

Supplement: Supplementary file 1 [file Presentation1.pptx]
