# Supplementary material for: Skeletal phenotypes in postmenopausal women affected by primary hyperparathyroidism
Source: Front Endocrinol (Lausanne). 2024 Oct 29;15:1475147. doi: 10.3389/fendo.2024.1475147 (PMC11558525; doi:10.3389/fendo.2024.1475147)
Supplement: Supplementary file 2 [file Table1.docx]

| **Parameters** | **Series 1**  **Milan** | **Series 2**  **Pisa** | **Series 3**  **Cuneo** | **p** |
| --- | --- | --- | --- | --- |
| n | 244 | 131 | 128 | - |
| Age (years) | 66.0 (61.0, 75.0) | 68.0 (61.0, 75.0) | 67.0 (61.0, 72.0) | 0.6905 |
| BMI (kg/m^2^) | 25.0 (22.3, 28.7) | 25.6 (23.2, 28.3) | 24.0 (22.0, 27.7) | 0.1053 |
| 25OHD (ng/ml) | 33.0 (25.3, 42.0) | 31.4 (24.7, 36.3) | 23.5 (15.0, 33.8)*,** | **<0.0001** |
| PTH (fold change) | 1.70 (1.33, 2.57) | 1.88 (1.45, 2.45) | 1.81 (1.42, 3.05) | 0.2553 |
| Ionized Ca^2+^ (mmol/L) | 1.40 (1.35, 1.48) | 1.45 (1.40, 1.52)* | 1.40 (1.35, 1.47)** | **<0.0001** |
| Total Ca^2+^ (mg/dl) | 10.7 (10.3, 11.2) | 10.9 (10.5, 11.4) | 11.0 (10.7, 11.4)* | **<0.0001** |
| UCa^2+^ (mg/24h) | 278.5 (197.0, 377.6) | 312.0 (200.0, 403.0) | 300.5 (188.0, 378.8) | 0.7364 |
| UCa^2+^ (mg/kg/24h) | 4.57 (3.05, 6.04) | 4.50 (2.9, 6.13) |  | 0.8560 |
| eGFR (ml/min) | 84.8 (68.3, 93.1) | 86.6 (77.0, 96.0) | 80.7 (73.1, 97.9) | 0.1245 |
| Phosphate (mg/dl) | 2.80 (2.5, 3.1) | 2.60 (2.33, 2.80)* | 2.90 (2.50, 3.20)** | **0.0001** |
| ALP (U/L) | 82.0 (67.0, 110.0) | 91.5 (76.5, 119.0) | 98.5 (79.5, 112.3)* | **0.0302** |
| BSAP (ng/ml) | 21.6 (13.2, 31.3) | 27.0 (17.0, 36.0) | 19.6 (13.1, 31.0)** | **0.0108** |
| Lumbar T-score | -2.40 (-3.20, -1.50) | -2.40 (-3.30, -1.40) | -2.70 (-3.40, -1.93) | 0.1179 |
| Neck T-score | -2.29±0.94 | -2.31±0.88 | -2.04±1.01*,** | **0.0271** |
| Fractured women (%) | 34.8 | 17.6* | 31.3** | **0.0019** |
| Kidney diseases (%) | 38.5 | 22.1* | 27.3* | **0.0026** |
| Hypertension (%) | 47.1 | 52.6 | 43.7 | 0.3447 |
| Diabetes (%) | 7.8 | 6.1 | 9.4 | 0.6165 |

*p<0.05 vs series 1; **p<0.05 vs series 2.

n, number of fragility fractures; BMD, bone mineral density; BMI, body mass index; 25OHD, 25hydroxyvitamin D; PTH, parathormone; eGFR, estimated glomerular filtration rate; ALP, alkaline phosphatase; BSAP, bone specific alkaline phosphatase. PTH is expressed as fold change of the upper limit of the reference range
